# Degenerative characteristics of multifidus at different vertebral levels of scoliosis in patients with degenerative lumbar scoliosis and relationship of these degenerative characteristics with surface electromyography activity

**DOI:** 10.1186/s12891-022-06038-6

**Published:** 2022-12-08

**Authors:** Hongru Xie, Qin Zhang, Jianan Liu, Yinchuan He, Zepei Zhang, Lin Meng, Jun Miao

**Affiliations:** 1 Yuncheng Central Hospital, NO. 3690 Hedong East Road, Yuncheng, 044000 Shanxi Province China; 2 Graduate School, Tianjin Medical University, Tianjin, China; 3 Department of Spine Surgery, Tianjin Hospital of Tianjin University, NO. 406 Jiefang South Road, Hexi District, Tianjin, 300210 China; 4 Academy of Medical Engineering and Translational Medicine, Tianjin University, NO.92 Weijin Road, Nankai District, Tianjin, 300210 China

**Keywords:** Degenerative lumbar scoliosis, Multifidus, Fat infiltration, Cross-sectional area, Surface electromyography

## Abstract

**Background:**

Previous studies have investigated the imaging changes of the paravertebral muscles (PVM) in patients with degenerative lumbar scoliosis (DLS); however, most of these studies focused on the apical vertebra (AV) level or compared with the average of the whole-segment measurement. In this study, magnetic resonance imaging (MRI) combined with surface electromyography (sEMG) was used to analyze the degenerative characteristics of the multifidus (MF) in patients with DLS at three levels of scoliosis (upper end vertebra [UEV], AV, and lower end vertebra [LEV]).

**Methods:**

This is a prospective cross-sectional study. Forty patients with DLS (DLS group) and 40 healthy individuals without lumbar scoliosis (control group) were evaluated. The percentage of fat infiltration area (%FIA) and muscle relative cross-sectional area (rCSA) of the MF at the three levels of scoliosis were measured on MRI, whereas the sEMG activity of the participants in both groups was recorded during action tasks. The imaging parameters, sEMG activity, and relationship between them were analyzed.

**Results:**

In the control group, there were no significant differences in the %FIA, rCSA, or sEMG activation of the MF between the bilateral sides at the three measured levels. In the DLS group, measurement of the MF at the UEV and AV levels showed that the %FIA was larger on the concave than convex side and that the rCSA was smaller on the concave than convex side, but there was no difference between the two parameters at the LEV level. In the standing flexion and extension tasks, the sEMG activation of the MF was higher on the concave than convex side. The coronal Cobb angle was correlated with the difference in the measurement data of the MF at the UEV and AV levels, but not with the difference at the LEV level.

**Conclusion:**

The MF in patients with DLS has the same degenerative features at the UEV and AV levels, with the concave side showing greater degeneration; however, there are different degenerative manifestations at the LEV level. The MF degeneration at the UEV and AV levels is the result of spinal deformity, while the muscle changes at the LEV level are more consistent with natural degeneration.

## Background

Degenerative lumbar scoliosis (DLS) is a spinal deformity due to lumbar degeneration after skeletal maturity, and its incidence increases gradually with age [1–3]. It has now become a common disease that threatens the health of the advanced-age population. DLS is a complex three-dimensional deformity involving coronal scoliosis, horizontal rotation of the vertebrae, and sagittal deformity. Although many studies have concentrated on DLS, the pathological mechanism (especially the role of the paravertebral muscles [PVM] in the development of scoliosis) remains a focus of debate among scholars.

Many scholars have conducted research on the PVM, including muscle tissue biopsy, biochemical index detection, and imaging analysis [4–6]. Numerous studies have suggested that spinal deformity may be related to PVM degeneration [4]. However, most previous studies focused on comparison of the apical vertebra (AV) level or the average measurement of the entire lumbar spine [7, 8]. No studies have involved comparisons among different levels of scoliosis (upper end vertebra [UEV], AV, and lower end vertebra [LEV]). In contrast, the present study is the first to distinguish the characteristics of PVM degeneration according to different levels of scoliosis, and the imaging parameters of the multifidus (MF) at the upper end vertebra (UEV) and lower end vertebra (LEV) levels were included in the research. In addition, most previous research involved static morphological studies, and such studies cannot reflect the dynamic changes of muscle function. In the present study, magnetic resonance imaging (MRI) and surface electromyography (sEMG) were combined for the first time to measure the characteristics of static degeneration (imaging parameters) and dynamic functional changes (muscle activation) and analyze their correlation.

Therefore, the aim of this study was to use MRI and sEMG to quantitatively evaluate the degenerative characteristics of the MF in patients with DLS at three levels of scoliosis (UEV, AV, and LEV) and to analyze the relationship between static measurement and dynamic function.

## Materials and methods

### Subjects

The DLS group comprised 40 patients with DLS diagnosed in Tianjin Hospital from March 2021 to June 2022. The inclusion criteria for this group were no history of scoliosis and a diagnosis of lumbar scoliosis with a Cobb angle of > 10° by examination; an age > 50 years; routine imaging examinations, including a standing full-length spine film and lumbar spine MRI; no nerve root lesions; and no history of treatment. The control group comprised 40 healthy individuals who received physical examinations in Tianjin Hospital from March 2021 to June 2022. The inclusion criteria for the control group were no scoliosis, an age of > 50 years, a standing full-length spine film and lumbar spine MRI, no radiculopathy, and no history of treatment.

The exclusion criteria for both groups were significant limitation of standing or lumbar extension and flexion; abnormal vertebral development, lumbar fracture, lumbar spondylolisthesis, or similar disorders, and a history of an operation on the lumbar spine, pelvis, or lower limb.

There were no significant differences in sex, age, height, weight, or body mass index between the DLS and control groups (Table 1).

**Table 1:** Demographic characteristics of the control group and DLS group (mean ± SD)

Variable	Control(*N* = 40)	DLS(*N* = 40)	Statistcs	*P* value
Gender(F/M)	24/16	28/12	χ^2^ = 3.31	*P* = 0.143
Age (years)	62.68 ± 7.97	64.9 ± 8.05	t = −0.543	*P* = 0.423
Height(m)	1.65 ± 0.04	1.64 ± 0.04	t = −1.841	*P* = 0.217
Weight (kg)	72.17 ± 9.22	69.23.0 ± 8.25	t = −1.364	*P* = 0.273
BMI	25.93 (16.27–29.39)^a^	25.33 ± 3.41	Z = -1.217	*P* = 0.321

^a^The BMI of the control group showed non-normal distribution, and the median was used to describe the data

### Imaging conditions

All subjects underwent radiography and MRI examinations using the same equipment. Lumbar X-ray examinations were performed using a 500-mA digital radiography system (AXIOM Aristos VX Plus; Siemens Healthineers, Erlangen, Germany) with 75 kV in the anteroposterior position and 85 kV in the lateral position using an automatic exposure control system. High-resolution 1.5 T MRI (MAGNETOM Essenza; Siemens Healthineers) was used for the lumbar spine with a slice width of 4 mm, acquisition matrix of 256 × 256, T1-weighted imaging repetition time of 587 ms (echo time of 11 ms), and T2-weighted imaging repetition time of 3000 ms (echo time of 93 ms).

### Imaging evaluation

**Fig. 1:**
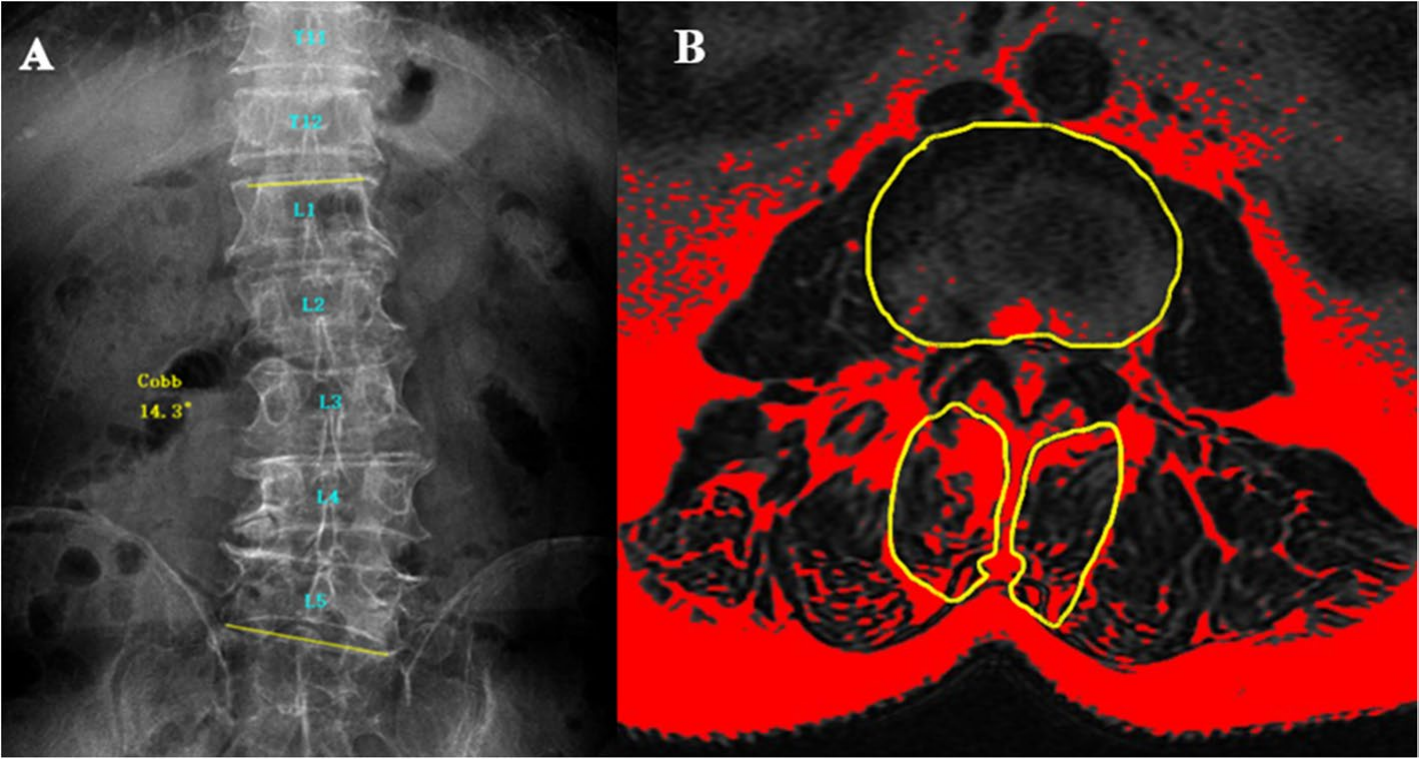
Images of a patient with left convex DLS. **A** The UEV is the L1 vertebra, the AV is the L3 vertebra, the LEV is the L5 vertebra, and the coronal Cobb angle is 14.3°. **B** Fat tissue in MRI is shown in red using the threshold technique. Three regions of interest were manually marked: the boundary of the bilateral MF and intervertebral disc

In the DLS group, the Cobb angle between the superior endplate of the UEV and the inferior endplate of the LEV was measured on the coronal plane of the lumbar spine. Thirty patients in the DLS group had left convex scoliosis and 10 patients had right convex scoliosis, and the mean coronal Cobb angle was 16.81° ± 6.24° (Fig. 1A).

T2 sequence images were selected for measurement, and three T2-weighted imaging images were taken at each disc level from T12/L1 to L5/S1, and intermediate slices were selected as the research object. Using ImageJ software v2.1.4.7 (National Institutes of Health, Bethesda, MD, USA), the region of interest was determined by manually marking the muscle boundaries. The inferior disc levels of the UEV, AV, and LEV were selected for measurement in the DLS group, and the inferior disc levels of the L1, L3, and L5 vertebrae were selected in the control group.

To reduce the effect of body size, height, and weight on muscle parameters, the ratio of the cross-sectional area (CSA) to the same-level disc CSA was used as the relative CSA (rCSA) [9]. The fat tissue in the 8-bit image was shown in red using the threshold technique [7] (Fig. 1B). The red area in the CSA of the MF was measured as the fat infiltrated area (FIA), and the percentage of FIA in the CSA of the MF was calculated as the percentage of FIA (%FIA) [4].

**Fig. 2:**
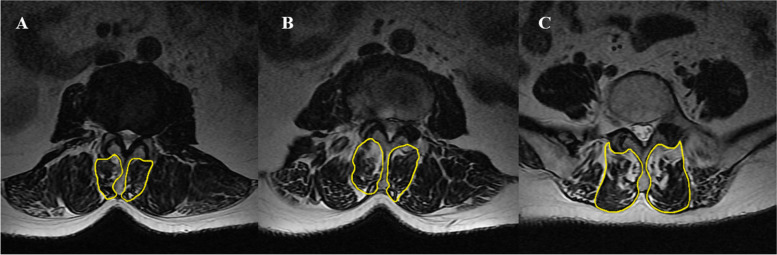
Images of patients with left convex DLS. **A** UEV level. The %FIA on the concave side was 34.36% and the rCSA was 23.45%. The %FIA on the convex side was 16.78% and the rCSA was 29.82%. **B** AV level. The %FIA on the concave side was 31.72% and the rCSA was 22.64%. The %FIA on the convex side was 22.78% and the rCSA was 27.13%. **C** LEV level. The %FIA on the concave side was 33.04% and the rCSA was 53.89%. The %FIA on the convex side was 34.40% and the rCSA was 54.31%

To facilitate comparison of the imaging parameters (rCSA and %FIA) of the MF on the convex and concave sides, the CSA difference index (CDI) and FIA difference index (FDI) were calculated. The following formulas were used: CDI = rCSA on the concave side − rCSA on the convex side, and FDI = %FIA on the concave side − %FIA on the convex side. The CDI and FDI are considered to represent the asymmetric degree of MF degeneration on the convex and concave sides [8, 10]. The DLS group showed obvious asymmetry in the degeneration of the MF on the convex and concave sides (Fig. 2).

### Reliability of the method

Muscle measurements on MRI were independently assessed by two examiners. Intraobserver repeatability and interobserver reliability were calculated by one-way analysis of variance and calculation of the intraclass correlation coefficient. According to the Fleiss criterion [11], an intraclass correlation coefficient of less than ±0.40 indicates poor reliability, ±0.40 to 0.75 indicates fair or good reliability, and ± 0.75 to 1.00 indicates excellent reliability. All data were measured with excellent intraobserver and interobserver reliability (Table 2).

**Table 2:** The correlation coefficients for intraobserver repeatability and interobserver reliability for both parameters were > 0.9, indicating that the measurements were reliable

Parameter	Level	intraobserver	interobserver
**%FIA**	**UEV**	0.962	0.981
	**AV**	0.973	0.967
	**LEV**	0.992	0.937
**rCSA**	**UEV**	0.977	0.964
	**AV**	0.984	0.979
	**LEV**	0.974	0.980

### sEMG measurements

**Fig. 3:**
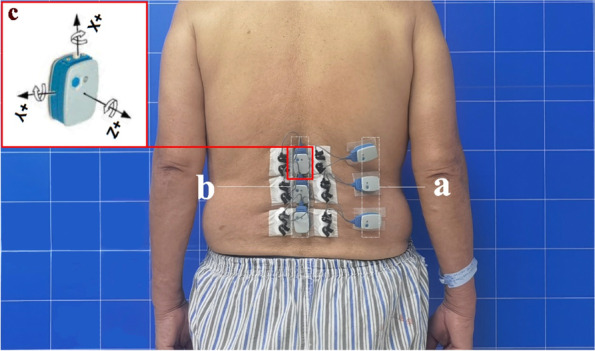
Six bipolar electrodes were placed at three levels (UEV, AV, and LEV) of the bilateral MF. After confirmation by X-ray, the sEMG sensors and the inertial measurement unit (IMU) were placed in the appropriate areas. **a** The three sEMG sensors on the right side. **b** Left sEMG sensors, each integrated with an IMU, were placed at the corresponding positions of the spinous processes (L1, L3, and L5). **c** Three axes of the IMU

An Ultium sEMG system (Noraxon USA, Scottsdale, AZ, USA) was used to record sEMG signals at a sampling rate of 2000 Hz. According to the European sEMG recommended standards [12], after skin preparation, Ag/AgCl bipolar circular surface electrodes (Shanghai, China) (44 × 22 mm; electrode spacing, 18 mm) prepared from conductive gel were placed on the bilateral MF muscle bellies at the UEV, AV, and LEV levels in the DLS group and at the L1, L3, and L5 levels in the control group (Fig. 3), consistent with the direction of the muscle fibers [13, 14].

### Tasks and procedures

**Fig. 4:**
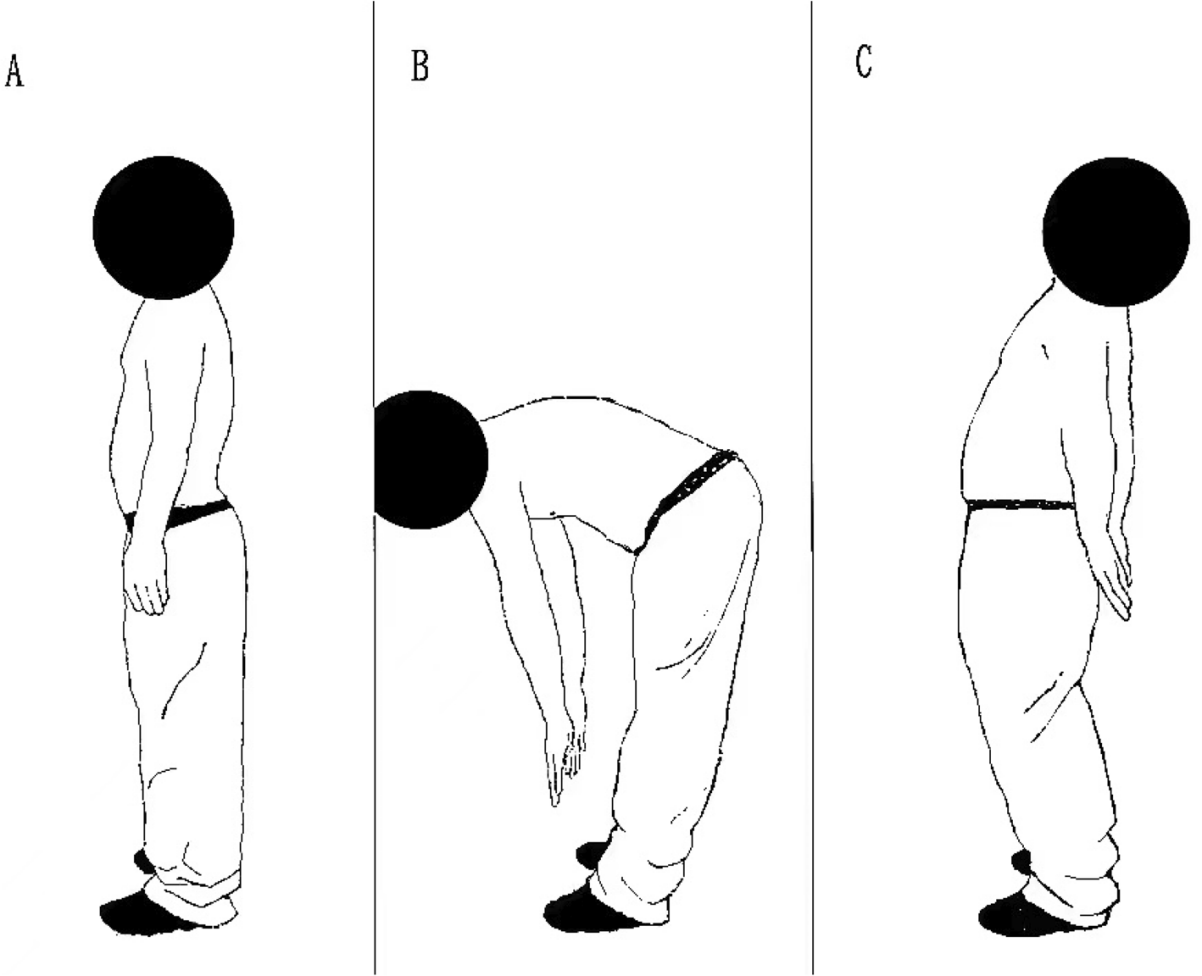
One subject performing the movement task. **A**: Standing still task. **B**: Maximum standing flexion. **C**: Maximum standing extension

The movement tasks included standing still, maximum standing flexion, and maximum standing extension. The subjects were asked to stand for 5 seconds in the resting state and then maintain maximum flexion and maximum extension for 5 seconds, and the sEMG signals in the three movement tasks were analyzed. Each subject was tested three times, and the average result of the three tests was used for further analysis (Fig. 4).

Before the formal measurement, the subjects were instructed to perform lumbar extension and flexion activities to familiarize themselves with the experimental process, avoiding participation of the pelvis and lower limbs as much as possible. To prevent fatigue, the patients had a 1-minute rest period between any two consecutive tests.

### Inertial measurement unit (IMU)

**Fig. 5:**
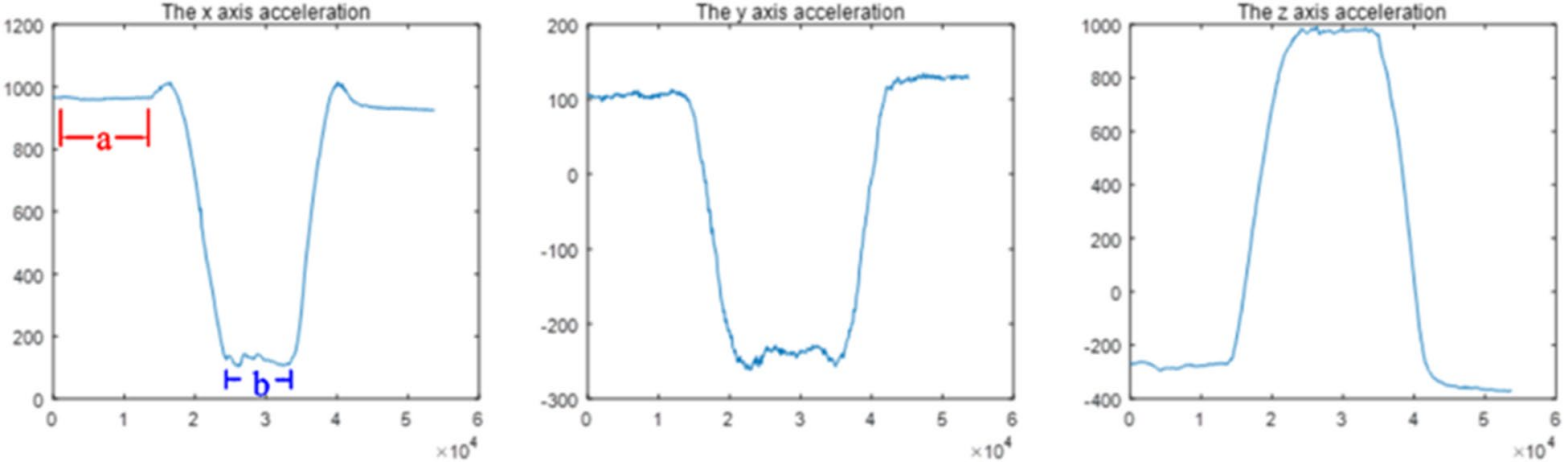
Typical processed images of three sets of motion signals acquired by the IMU: acceleration along the vertical axis (x-axis), horizontal axis (y-axis), and anteroposterior axis (z-axis). **a** Standing still phase. **b** Maximal standing flexion/extension maintenance phase. The X-axis is the data acquisition frequency (Hz), and the Y-axis is the acceleration of motion (mg)

The three sEMG sensors on the left side were integrated with the IMU [15, 16] and fixed on the spinous processes at the L1, L3, and L5 levels using double-sided tape to collect motion signals (Figs. 3,5).

### sEMG signal processing

The raw sEMG signal was processed using a Butterworth bandpass filter (30–500 Hz), and baseline noise was removed. Using the kinematic data acquired simultaneously by the IMU, the 3-second sEMG data with smooth movements were selected for analysis. The root mean square was calculated using MATLAB software (MATLAB R2020a; MathWorks, Natick, MA, USA) and normalized to the ratio to the maximal voluntary isometric contraction (MVIC), or %MVIC, representing the degree of muscle activation. Larger values indicate higher levels of muscle activation, allowing comparison of different levels of muscle activity [17].

### Statistical analysis

Statistical analysis was performed using SPSS 25.0 (IBM Corporation, Armonk, NY, USA). Measurement data are presented as mean ± standard deviation. All data were analyzed by descriptive analysis and normality tests. Non-parametric tests were used to compare the demographic data of the two groups. Multifactorial analysis of variance was used to evaluate the effects of influencing factors (experimental grouping, left and right/convex and convex, and different levels of scoliosis) on imaging parameters and sEMG activation. Pearson’s correlation test was used to analyze the relationship between the coronal Cobb angle and the radiographic and sEMG data differences (degenerative asymmetry). A *P* value of < 0.05 was considered to indicate a statistically significant difference.

## Results

### Imaging parameters of MF at the same level (left and right, convex and concave) in the two groups

In the control group, there was no significant difference in %FIA or rCSA of the bilateral MF at the three measurement levels. In the DLS group, at the UEV and AV levels, the %FIA was greater on the concave than convex side and the rCSA was smaller on the concave than convex side, indicating that the degree of degeneration was greater on the concave than convex side. At the LEV level, there was no significant difference in the two imaging parameters (Table 3). Thus, in the DLS group, asymmetry of the degeneration on the convex and concave sides was present at the UEV and AV levels but not at the LEV level.

**Table 3:** MRI measurements of MF in both groups

Parameter	Level	Control-Right	Control-Light	***P*** value	Level	DLS-concave	DLS-convex	***P*** value
**%FIA**	**L1**	0.262 ± 0.098	0.274 ± 0.094	0.077	**UEV**	0.341 ± 0.112	0.265 ± 0.077	0.000*
	**L3**	0.258 ± 0.074	0.254 ± 0.078	0.587	**AV**	0.330 ± 0.099	0.260 ± 0.079	0.000*
	**L5**	0.303 ± 0.070	0.304 ± 0.064	0.971	**LEV**	0.302 ± 0.091	0.318 ± 0.095	0.112
**rCSA**	**L1**	0.179 ± 0.061	0.182 ± 0.062	0.456	**UEV**	0.241 ± 0.116	0.261 ± 0.104	0.009*
	**L3**	0.345 ± 0.110	0.350 ± 0.112	0.293	**AV**	0.320 ± 0.142	0.360 ± 0.152	0.001*
	**L5**	0.535 ± 0.168	0.538 ± 0.157	0.774	**LEV**	0.516 ± 0.205	0.496 ± 0.198	0.054

Control-Right: The right multifidus in the Control group. Control-Light: The left multifidus in the Control group. DLS-concave: The concave multifidus in the DLS Group. DLS-convex: The convex multifidus in the DLS Group

### Imaging parameters of the two groups at different measurement levels

**Fig. 6:**
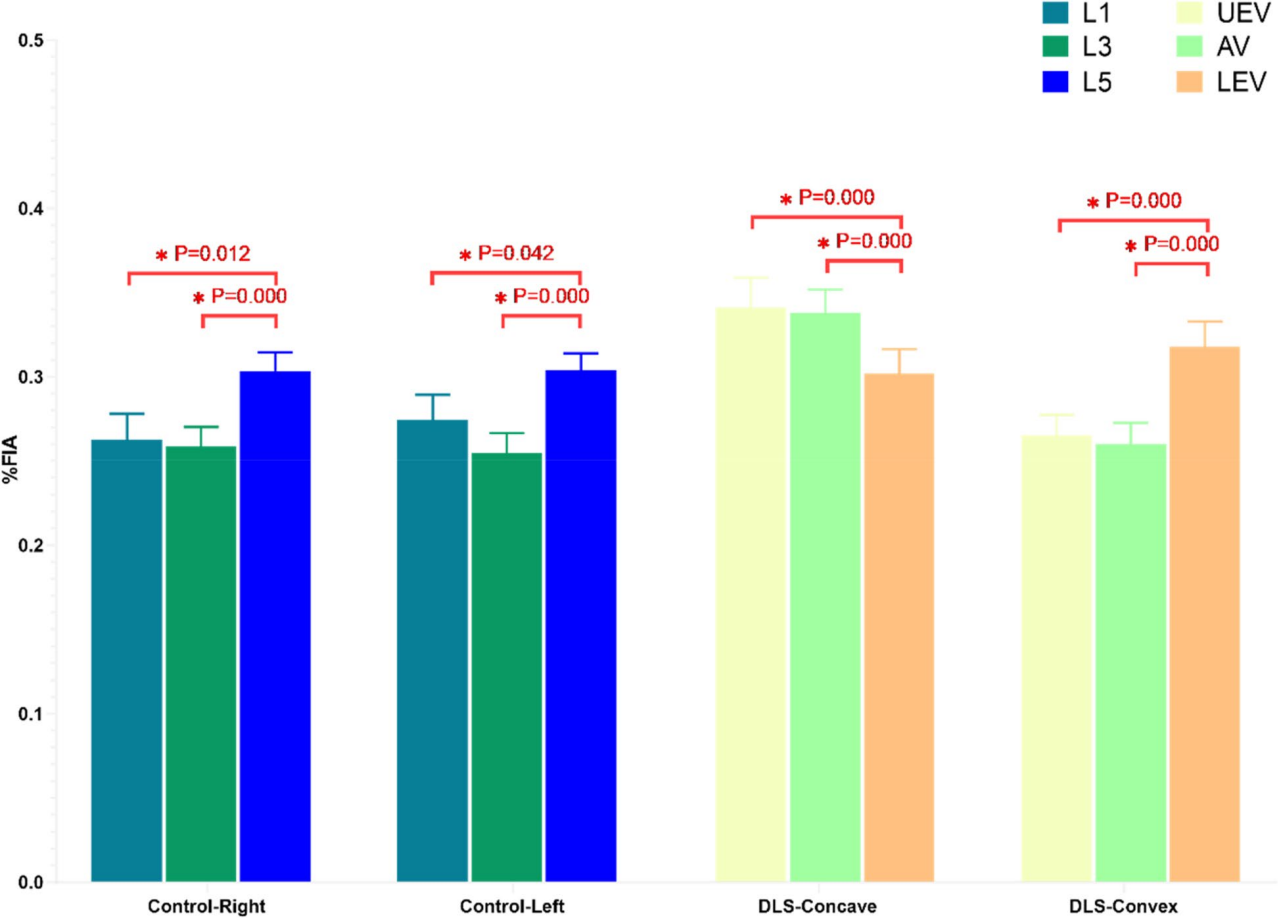
%FIA of the control and DLS groups at three measurement levels. Control-Right: The right multifidus in the Control group. Control-Light: The left multifidus in the Control group. DLS-concave: The concave multifidus in the DLS Group. DLS-convex: The convex multifidus in the DLS Group

In the control group, the %FIA at the L1 level was not significantly different from that at the L3 level, but both were significantly lower than that at the L5 level (Fig. 6), which is consistent with previous literature. In the DLS group, the %FIA on the concave side at the UEV level was not significantly different from that at the AV level (*P* = 0.543), but both were higher than that at the LEV level (Fig. 6). Additionally, the %FIA on the convex side at the UEV level was not significantly different from that at the AV level (*P* = 0.141), but both were lower than that at the LEV level (Fig. 6). The rCSA in both groups gradually increased as the lumbar vertebral level decreased.

### Degree of sEMG activation in the two groups

**Fig. 7:**
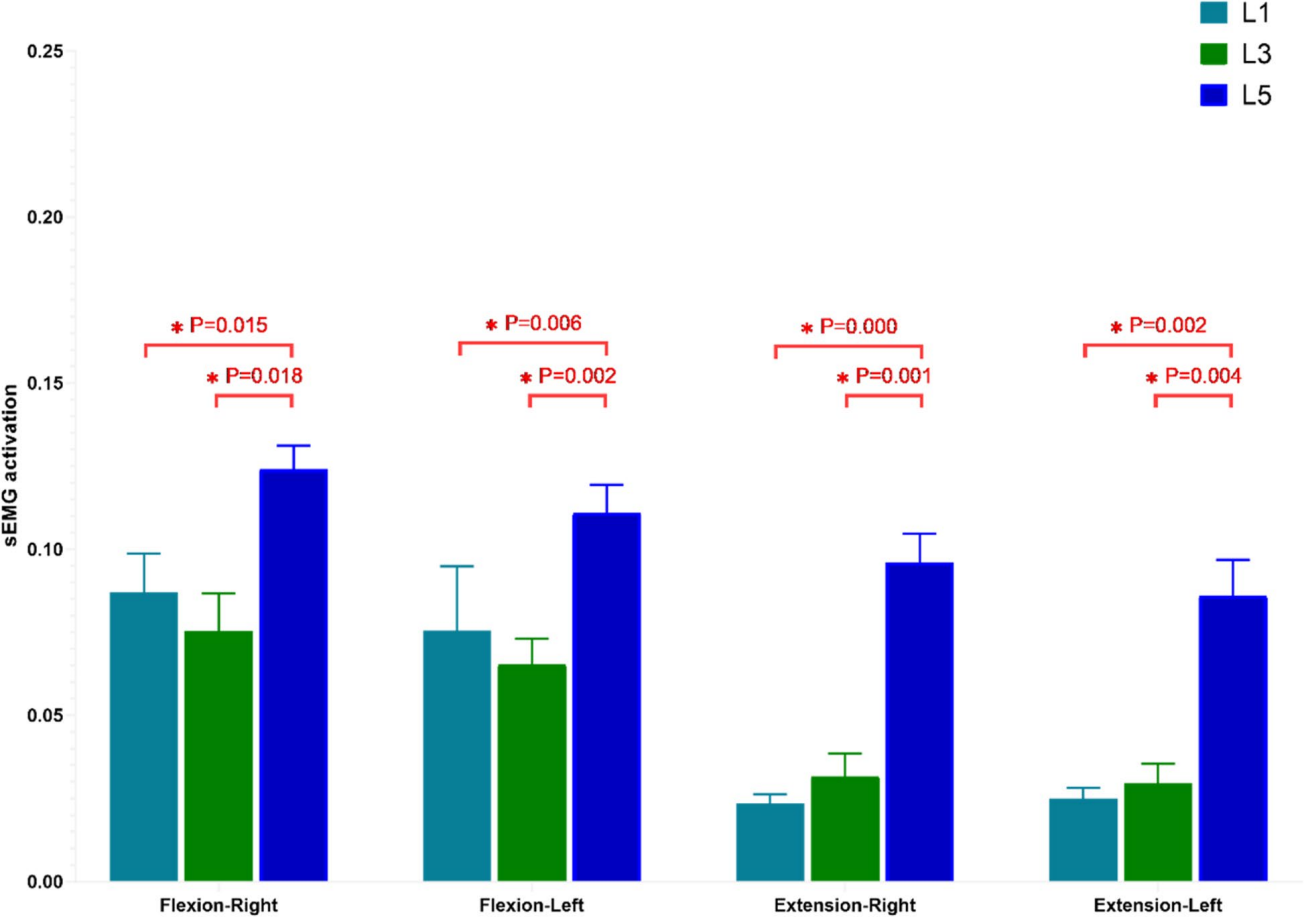
sEMG activation in the control group at three measured levels. Flexion: maximum standing flexion. Extension: maximum standing extension

**Fig. 8:**
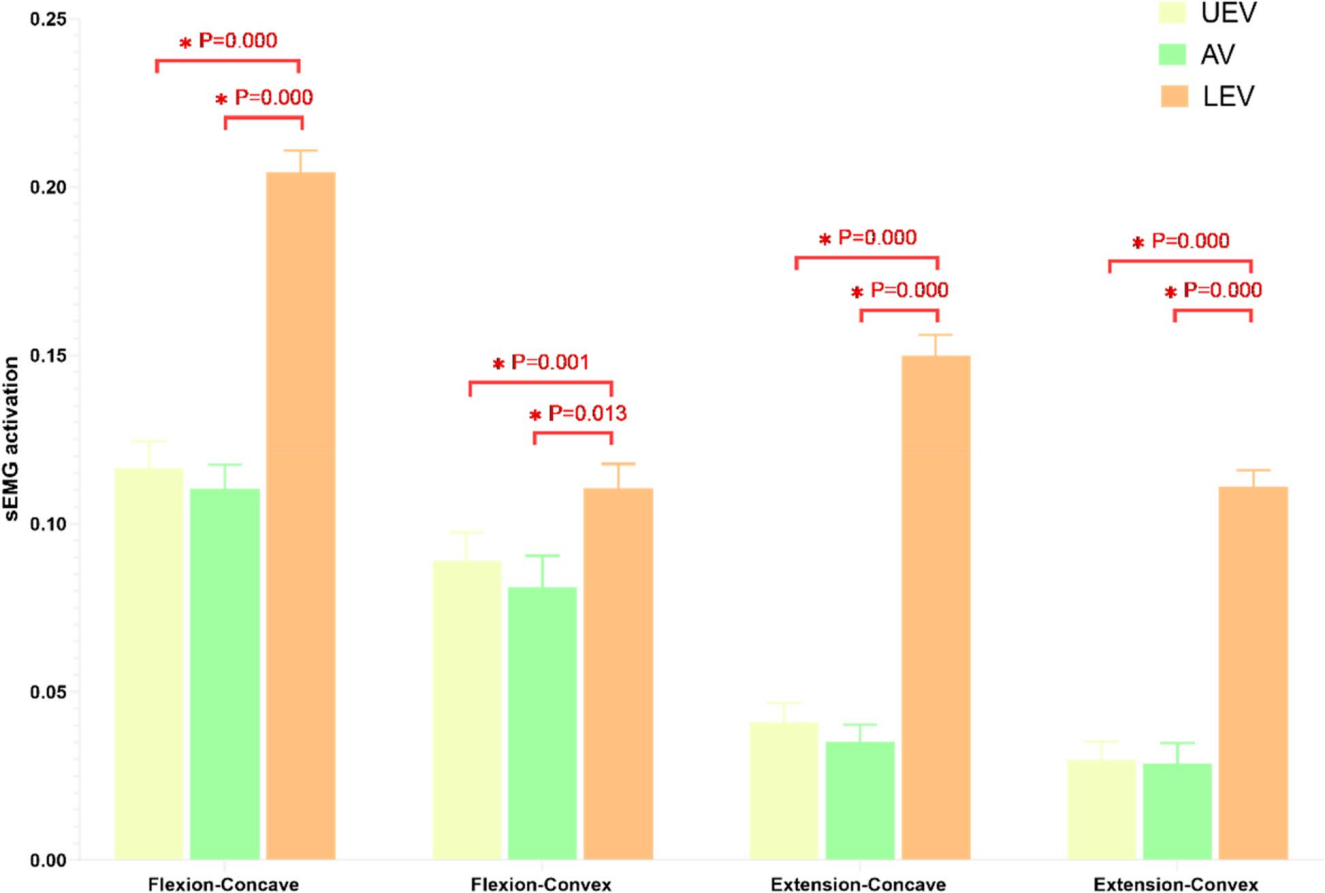
sEMG activation in the DLS group at three measured levels. Flexion: maximum standing flexion. Extension: maximum standing extension

In the control group, there were no significant differences in the sEMG activation of the bilateral MF in the three movement tasks. In the DLS group, sEMG activation was not significantly different between the convex and concave sides in the standing still task. In the maximum standing flexion and maximum standing extension tasks, however, the three measurement levels showed that the sEMG activation was greater on the concave than convex side (Table 4). Among the different measurement levels in both the DLS and control groups, the sEMG activation did not differ between the UEV(L1) and AV(L3) levels, but both were lower than that at the LEV(L5) level (Figs. 7,8).

**Table 4:** sEMG measurements of MF in both groups

Movement task	Level	Control-Right	Control-Light	***P*** value	Level	DLS-concave	DLS-convex	***P*** value
**standing still**	**L1**	0.057 ± 0.036	0.049 ± 0.018	0.526	**UEV**	0.057 ± 0.029	0.062 ± 0.027	0.298
	**L3**	0.05 ± 0.032	0.054 ± 0.029	0.980	**AV**	0.051 ± 0.032	0.058 ± 0.040	0.262
	**L5**	0.083 ± 0.031	0.078 ± 0.039	0.451	**LEV**	0.055 ± 0.032	0.056 ± 0.030	0.877
**maximum standing flexion**	**L1**	0.087 ± 0.041	0.076 ± 0.067	0.558	**UEV**	0.117 ± 0.049	0.089 ± 0.053	0.000*
	**L3**	0.075 ± 0.040	0.065 ± 0.026	0.366	**AV**	0.110 ± 0.045	0.081 ± 0.059	0.005*
	**L5**	0.124 ± 0.026	0.111 ± 0.029	0.294	**LEV**	0.205 ± 0.041	0.111 ± 0.046	0.000*
**maximum standing extension**	**L1**	0.024 ± 0.009	0.024 ± 0.011	0.743	**UEV**	0.041 ± 0.033	0.030 ± 0.031	0.017*
	**L3**	0.032 ± 0.023	0.030 ± 0.019	0.793	**AV**	0.035 ± 0.028	0.029 ± 0.035	0.039*
	**L5**	0.041 ± 0.017	0.037 ± 0.036	0.616	**LEV**	0.150 ± 0.035	0.111 ± 0.029	0.000*

Control-Right: The right multifidus in the Control group. Control-Light: The left multifidus in the Control group. DLS-concave: The concave multifidus in the DLS Group. DLS-convex: The convex multifidus in the DLS Group

### Correlation between the difference in the measurement index and the coronal Cobb angle

At the UEV and AV levels, the FDI and CDI were positively correlated with the coronal Cobb angle (Table 5); however, in terms of the degree of sEMG activation, only the difference at the AV level was positively correlated with the coronal Cobb angle (Table 6). At the LEV level, neither imaging parameters nor sEMG activation were associated with the coronal Cobb angle.

**Table 5:** Correlation between the coronal Cobb angle and the difference index of imaging parameters

Index	UEV -FDI	AV -FDI	UEV -CDI	AV -CDI
**coronal Cobb angle**	***r*** **= 0.413**	***r*** **= 0.488**	***r*** **= 0.381**	***r*** **= 0.317**
	***p*** = 0.031^*^	***p*** = 0.001^**^	***p*** = 0.015^*^	***p*** = 0.046^*^

**Table 6:** Correlation of the coronal Cobb angle with the difference of sEMG activation between the convex and concave sides

Index	maximum standing flexion			maximum standing extension		
	UEV	AV	LEV	UEV	AV	LEV
**coronal Cobb angle**	*r* = 0.039	***r*** **= 0.655**	*r* = 0.192	*r* = 0.158	***r*** **= 0.472**	*r* = 0.320
	*p* = 0.812	***p*** = 0.000^**^	*p* = 0.235	*p* = 0.380	***p*** = 0.006^**^	*p* = 0.069

## Discussion

The PVM play a crucial role in the stability and motor function of the lumbar spine and are associated with various lumbar spine diseases and postoperative complications [18, 19]. The MF is sensitive to pathological changes and is the muscle that shows the most obvious and most common degeneration among the lumbar PVM [20, 21]. Therefore, we focused on the MF in the present study.

MRI can clearly display muscle mass and intramuscular fat, show the difference between fat and muscle signal strength, and facilitate measurement of changes in muscle size and infiltration of muscle by fat tissue. Therefore, MRI is an ideal imaging method for evaluating the morphology of lumbar PVM and can reflect the static functional changes of PVM [6, 8]. As a response of human neuromuscular activity, sEMG signals represent the change in muscle dynamic function and can be used to analyze the correlation between PVM and scoliosis [22].

Most previous studies used the %FIA or rCSA at the AV level to compare the characteristics of muscle degeneration between different groups. No relevant reports have described the imaging manifestations of the PVM at different levels of scoliosis in patients with DLS. In this study, we used the same threshold technique for muscles at all measured levels, enabling the results to be compared between muscles [23]. In addition, MRI can provide indicators of imaging changes, and sEMG can reflect changes in muscle electrical activity; however, the relationship between imaging parameters and muscle dynamic electrical activity is unclear. We combined the two to help further clarify the characteristics of PVM degeneration in patients with DLS.

### Degenerative features of the MF at the same level (left and right, convex and concave)

In our study, MF degeneration on the convex and concave sides in patients with DLS showed asymmetry, but this asymmetry had different characteristics at different levels of scoliosis (UEV, AV, and LEV).

PVM degeneration is characterized by an increase in fat deposition or a decrease in muscle size [6, 21, 24]. We found that in the DLS group, at the UEV and AV levels, the imaging parameters (%FIA and rCSA) of the MF were significantly different between the convex and concave sides and that the decrease in the rCSA on the concave side was accompanied by an increase in the %FIA. These findings indicated asymmetrical degeneration with greater degeneration on the concave than convex side. In contrast, there was no such asymmetry in the control group. This is consistent with the results of previous studies. Xie [7] found that fat infiltration of the MF was significantly greater on the concave than convex side in patients with DLS, but the index used was the average fat infiltration rate of the whole segment of the lumbar spine. Shafaq [4] found that the diameter of muscle fibers and the number of muscle cell nuclei on the concave side of the MF were reduced and that the muscle CSA was smaller on the concave than convex side; however, the comparison was made with lumbar spinal stenosis at the same spinal level, and the changes were not classified according to the level of scoliosis.

The appearance of this degenerative asymmetry is thought to be the result of biomechanical compensation. Because of the large load on the lumbar extensor muscle group, scoliosis leads to different forces on both sides, and the tension load is greater on the convex than concave side [8, 10]. The high load on the convex side leads to compensatory hyperplasia and hypertrophy as well as inhibition of adipocyte differentiation [25], while atrophy and steatosis appear on the concave side. Therefore, the purpose of the compensatory hypertrophy of the MF is to maintain the coronal balance of the spine and reduce the inclination of the spine to the concave side, while the muscle atrophy on the concave side may be related to increased fat infiltration.

In the present study, the sEMG results of the same level showed that the DLS group exhibited a greater degree of sEMG activation on the concave than convex side at all three measurement levels. This occurred because weaker or more degenerated PVM require higher sEMG activation to maintain spine stability and balance [26], which is consistent with the greater degree of degeneration on the concave than convex side in the imaging results of the present study. However, the patients with DLS did not show this asymmetry in convex/concave degeneration at the LEV level. The LEV level may have biomechanical, anatomical, and compensatory factors that differ from those at the UEV and AV levels.

### Degenerative features of the MF at different levels (UEV, AV, and LEV)

We found that fatty infiltration of the MF was greater at the LEV level than at the UEV and AV levels. This is consistent with previous studies showing that the rate of muscle degeneration differs at different levels of the lumbar spine. PVM degeneration starts at the lower lumbar level and gradually progresses upward [27]. Hyun [6] found that the degeneration and fatty infiltration of the PVM was more severe at the lower than upper lumbar level, which is the same conclusion reached by Xia [28]. Greater force is exerted in closer proximity to the lower lumbar vertebrae, increasing the likelihood of muscle degeneration.

Notably, our study also showed that at the LEV level, there were no significant differences in imaging parameters (%FIA and rCSA) of the MF between the convex and concave sides, and the asymmetry of degeneration disappeared. According to the sEMG results, more sufficient sEMG activation is observed at lower levels of the MF, which may be related to the cantilever mechanism [27, 29]. During daily extension and flexion activities, mobilization of the muscles at the LEV level was more adequate than that at the UEV and AV levels, especially on the concave side. This may eventually correct the muscle atrophy and steatosis, resulting in the disappearance of the asymmetry of lower vertebral degeneration. In contrast, the muscles at the UEV and AV levels were not sufficiently activated during exercise and cannot correct this degenerative feature.

### Relationship between degenerative features of the MF and the coronal Cobb angle

Previous studies have shown that asymmetry of PVM degeneration is positively correlated with the coronal Cobb angle, with a more severe scoliosis deformity being associated with greater asymmetry of PVM degeneration. This was discussed in reports by Yagi [30], Tang [10], Ding [31], and Xia [28]. Thus, the asymmetry of the PVM in patients with DLS reflects the severity of the lumbar coronal imbalance [31].

However, our study showed that the imaging differential index at the UEV and AV levels was related to the coronal Cobb angle, whereas the sEMG difference showed a correlation only at the AV level; at the LEV level, regardless of the difference in imaging parameters or sEMG activation, was neither was related to the degree of spinal coronal deformity. It is evident that the effect of lumbar scoliosis on muscles occurs only at the UEV and AV levels, not at the LEV level. Therefore, the asymmetry of MF degeneration at the UEV and AV levels is caused by coronal scoliosis, whereas the degeneration characteristics of MF at the LEV level are more consistent with the natural degeneration pattern of the PVM and are not associated with the degree of scoliosis.

### Study limitations

This study had two main limitations. First, previous cytological and pathological studies to support our results are lacking. Second, no subgroup analyses according to the severity of lumbar scoliosis were performed. Third, there is degeneration of intervertebral discs, endplates and facet joint in the DLS group, while the control group is relatively light, which may have an impact on the research results.

## Conclusions

The four main findings of this study are as follows. First, the degeneration of the MF on the convex and concave sides of patients with DLS showed asymmetry, but this asymmetry had different characteristics at different levels of scoliosis (UEV, AV, and LEV). Second, the MF at the UEV and AV levels had the same degeneration characteristics (the %FIA was greater on the concave than convex side, the rCSA was smaller on the concave than convex side, and the degeneration was greater on the concave than convex side), but this asymmetry of degeneration failed to be reflected at the LEV level. Third, the degeneration of the MF at the UEV and AV levels was related to the degree of scoliosis, while the degeneration of the MF at the LEV level was associated with the natural degeneration pattern. Finally, during lumbar extension and flexion, the greater degree of muscle activation at the lower levels of the MF may have ultimately led to the disappearance of degenerative asymmetry at the LEV level.

## Data Availability

The datasets used and/or analyzed during the current study are available from the corresponding author on reasonable requet.

## Abbreviations

DLS: Degenerative lumbar scoliosis
PVM: Paravertebral muscle
UEV: Upper end vertebra
AV: Apical vertebra
LEV: Lower end vertebra
MF: Multifidus
sEMG: Surface electromyography
FIA: Fat infiltration area
CSA: Cross-sectional area
MRI: Magnetic resonance image
RMS: Root mean square
rCSA: Relative cross-sectional area
T1WI: T1-weighted images
T2WI: T2-weighted images
ROI: Region of interest
IMU: Inertial measurement unit
MVIC: Maximal voluntary isometric contraction
